# Flavonoids, Thyroid Iodide Uptake and Thyroid Cancer—A Review

**DOI:** 10.3390/ijms18061247

**Published:** 2017-06-12

**Authors:** Carlos F. L. Gonçalves, Mariana L. de Freitas, Andrea C. F. Ferreira

**Affiliations:** 1 Carlos Frederico Lima Gonçalves, Laboratory of Endocrine Physiology, Instituto de Biofísica Carlos Chagas Filho, Universidade Federal do Rio de Janeiro, 21941-902 Rio de Janeiro, Brazil; fredenut@yahoo.com.br; 2 Mariana Lopes de Freitas, Laboratory of Endocrine Physiology, Instituto de Biofísica Carlos Chagas Filho, Universidade Federal do Rio de Janeiro, 21941-902 Rio de Janeiro, Brazil; marianalopes_ufrj@yahoo.com.br; 3 Andrea Claudia Freitas Ferreira, Laboratory of Endocrine Physiology, Instituto de Biofísica Carlos Chagas Filho, Universidade Federal do Rio de Janeiro, 21941-902 Rio de Janeiro, Brazil; 4 NUMPEX, Campus Duque de Caxias, Universidade Federal do Rio de Janeiro, Duque de Caxias, 25245-390 Rio de Janeiro, Brazil

**Keywords:** thyroid, cancer, flavonoid, phytochemical, sodium iodide symporter (NIS), iodide uptake, radioiodine therapy, proliferation, invasiveness

## Abstract

Thyroid cancer is the most common malignant tumor of the endocrine system and the incidence has been increasing in recent years. In a great part of the differentiated carcinomas, thyrocytes are capable of uptaking iodide. In these cases, the main therapeutic approach includes thyroidectomy followed by ablative therapy with radioiodine. However, in part of the patients, the capacity to concentrate iodide is lost due to down-regulation of the sodium-iodide symporter (NIS), the protein responsible for transporting iodide into the thyrocytes. Thus, therapy with radioiodide becomes ineffective, limiting therapeutic options and reducing the life expectancy of the patient. Excessive ingestion of some flavonoids has been associated with thyroid dysfunction and goiter. Nevertheless, studies have shown that some flavonoids can be beneficial for thyroid cancer, by reducing cell proliferation and increasing cell death, besides increasing NIS mRNA levels and iodide uptake. Recent data show that the flavonoids apingenin and rutin are capable of increasing NIS function and expression in vivo. Herein we review literature data regarding the effect of flavonoids on thyroid cancer, besides the effect of these compounds on the expression and function of the sodium-iodide symporter. We will also discuss the possibility of using flavonoids as adjuvants for therapy of thyroid cancer.

## 1. Introduction

Flavonoid is the name of a great group of phytochemical compounds of natural origin, composed by aromatic substances widely distributed in the vegetable kingdom. Flavonoids are not synthesized by human beings but we can obtain them through the diet [1].

Even though the role of the flavonoids in human metabolism is not clear, the ingestion of these compounds is about 23 to 34 mg daily, and can reach two grams daily depending on eating habits, since they can be found in many types of food and beverages, including fruits, grains, salads, wine, teas, etc. [2,3,4].

The use of flavonoids in therapy is broad and ancient, yet often empiric. The benefits of vegetable ingestion have been frequently attributed to the flavonoids, more than to other phytochemicals. In fact, there are studies showing pharmacological properties of flavonoids, such as antioxidant and anti-inflammatory, among others [1]. Thus, flavonoids are getting increasing attention from the pharmaceutical and food industries. Medicines and food supplements containing flavonoids have been suggested to treat circulatory disorders and hypertension, among other diseases [5,6].

Since 1958, it is known that flavonoids can affect thyroid function [7]. This effect is of particular concern in areas of low iodine intake, since the consumption of flavonoids could lead to endemic goiter and hypothyroidism [8]. Other works have shown that some flavonoids could impact not only thyroid hormone synthesis [9,10,11,12,13,14,15], but also thyroid hormone metabolism [15,16,17,18,19,20]. On the other hand, recent data have suggested that flavonoids could have beneficial effects on thyroid cancer. For example, myricetin [21], quercetin [22] and apigenin [23] have been shown to induce cell death in thyroid cancer cell lines. Furthermore, some flavonoids seem to increase iodide uptake in thyroid cell lines, such as myricetin [24] and apigenin [25], as well as in vivo thyroid iodide uptake [15]. The ability of thyroid cancer to uptake iodide is crucial to the efficacy of radioiodine therapy; however, part of the patients lose their capability to concentrate radioiodine, due to the dedifferentiation process during carcinogenesis [26]. Therefore, compounds able to increase iodide uptake in thyroid cancer could be useful as adjuvant in radioiodine therapy.

## 2. Sodium-Iodide Symporter

Sodium-iodide symporter (NIS) plays a key role in thyroid physiology, since it transports iodide into the thyroid gland, an element constituent of thyroid hormones [27,28]. In addition, NIS plays a central role in thyroid therapeutics, since the use of radioiodine is a safe and effective method for treatment of thyroid disease, especially thyroid cancer [26,27,28,29]. In most differentiated thyroid carcinomas, the ability to uptake iodide is maintained, so the treatment using the radionuclide 131I is often chosen as the main therapeutic approach after total thyroidectomy for complete ablation of remnant thyroid tissue [28]. However, in a portion of patients with thyroid cancer, the thyrocytes lose their ability to uptake iodide, due to dedifferentiation, making the radioiodine therapy ineffective [30]. Patients who have tumors with this characteristic are considered as a therapeutic challenge, since the treatment options become limited and often inefficient [26,31,32].

Sodium-iodide symporter is located in the basolateral membrane of the thyrocytes and transports iodide from the bloodstream into the cell [33]. The cloning of cDNAs from both human and rat *NIS* occurred in 1996, by different researcher groups [33,34]. Human *NIS* gene is located in chromosome 19p12-13.2 and encodes an integral membrane glycoprotein of 643 amino acids, which has 84% homology with the protein encoded by the mouse *NIS* gene [35,36]. NIS is a glycoprotein with thirteen transmembrane domains. The amino-terminus is extracellular while the carboxy-terminus is intracellular [37]. It is also a phosphoprotein, with most phosphorylation sites in the carboxy-terminal region. Phosphorylation in this region seems to be important for NIS targeting and localization in the basolateral membrane of the thyrocyte [38]. Although sodium-iodide symporter possesses three glycosylation sites, they do not seem to be important for NIS function, since it has been shown that *NIS* mutations in these sites do not affect the affinity for iodide [37].

**Figure 1:**
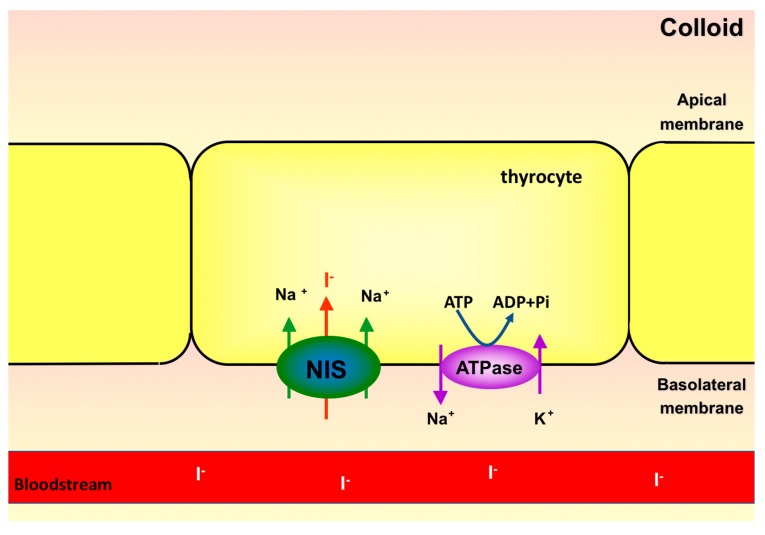
Transport of iodide into thyrocytes by sodium-iodide symporter (NIS).

NIS couples the transport of two sodium ions, favorable to electrochemical gradient, to an iodide ion, against its electrochemical gradient (Figure 1). The gradient favoring sodium entrance is generated by Na^+^/K^+^-ATPase, also found in the basolateral membrane of thyrocytes [36]. This ATPase transports 3 Na^+^ ions out and 2 K^+^ ions into the follicular cell, at the expense of ATP hydrolysis energy. Therefore, Na^+^/K^+^-ATPase is a primary active transporter, whereas NIS is considered a secondary active transporter [36]. Despite being able to transport other anions, NIS has high affinity for iodide. Selenocyanate, thiocyanate, ClO^3−^ and NO^3−^ can be transported by NIS and thus compete with iodide, decreasing intracellular iodide accumulation [39]. During the first experiments characterizing the transport of anions by NIS, it was observed that ClO^4−^ is 10–100 times more potent than thiocyanate as NIS inhibitor. Even though ClO^4−^ is not transported by NIS, it is able to block the transport of iodide, due to the high affinity of NIS for ClO^4−^ [27,39]. Similar to all other steps of thyroid hormone synthesis, NIS expression and activity are dependent on TSH stimulation [40], even in in vitro thyrocytes culture [41,42]. Removal of TSH from culture medium leads to reduction of cyclic adenosine monophosphate (cAMP) levels and iodide uptake in cultured Fisher rat thyroid cell line (FRTL-5) [43]. In addition to modulating the expression and function of NIS, TSH also regulates its subcellular distribution, being fundamental for the targeting and/or maintenance of NIS in the plasma membrane, where it is functional [44].

The main intracellular mechanism by which the thyrotrophic hormone regulates the expression of the sodium/iodide symporter involves the activation of adenylate cyclase, protein kinase A and CREB transcription factor [36,45]. Besides increasing the intracellular levels of cAMP, TSH can also act by activating other intracellular pathways, such as ERK-MEK and p38MAPK [46]. However, the actual importance of these pathways as modulators of the expression and activity of NIS is still controversial [46,47,48]. NIS expression and function can also be modulated by pathways activated by insulin and IGF-1 [49,50]. This pathway, which seems to have an inhibitory effect on NIS, involves the participation of phosphatidyl inositol-3-kinase (PI3K) [49,50] and the mechanistic target of rapamycin (mTOR), which has been described to participate in NIS modulation by our group [51]. AMP-activated kinase (AMPK) has also been shown to have a fundamental stimulatory role on the activity and expression of NIS [52,53,54].

## 3. Thyroid Cancer

Thyroid cancer is the most frequent malignant tumor of the endocrine system and corresponds to about 1% of all new cancers diagnosed in the United States [55]. The incidence of thyroid cancer is increasing and it is an important concern in public health. In Brazil, estimates from the Ministry of Health pointed to an amount of 1090 new cases for men and 5870 for women, with an estimated risk of 1.08 cases per 100,000 men and 5.70 cases per 100,000 women [56]. There are some environmental/lifestyle factors that increase the risk to develop thyroid cancer, e.g., radiation exposure [57], female gender [58], familial cases of thyroid cancer, and aging [59]. In addition, a recent review proposes that extreme living and working conditions, such as long-term exposure to gravity modifications, could impair thyroid function and/or lead to thyroid cancer [60].

It is known that thyroid tumors derivate from two distinct cellular populations: C cells and follicular cells, being basically divided in differentiated carcinomas, poorly differentiated carcinomas and undifferentiated carcinomas [61]. The differentiated thyroid carcinomas of follicular cells correspond to approximately 90% of thyroid carcinoma cases and are essentially classified as papillary or follicular. The most frequent histological type is the papillary thyroid carcinoma, which is more responsive to treatment, being usually associated with a good prognosis [61,62,63]. On the other hand, poor differentiated and undifferentiated thyroid carcinomas (e.g., anaplastic carcinoma) are more aggressive and lethal, but fortunately the prevalence is lower than that of differentiated thyroid carcinomas [55]. Thyroid follicular carcinoma is the second most common histological type, corresponding to almost 10% of all thyroid cancers. The progression of the disease is faster than in papillary carcinoma and there is a greater risk of distant metastasis, with lungs and bones being the most common metastatic sites [64]. Nevertheless, follicular carcinomas in general are responsive to treatment, although the prognosis is slightly worse than that of papillary carcinoma.

The genetic alterations most commonly found in differentiated carcinomas involve the mutation or rearrangement of genes encoding proteins involved in the MAP kinase pathway [65]. The most prevalent changes are BRAF mutations (29–70%) [66] and RET/PTC translocation (13–43%) in papillary carcinomas [61,66]. Activation of this pathway stimulates cell proliferation and its constitutive activation promotes tumorigenesis [67].

BRAF is a serine-threonine kinase that plays a role in signal transduction of the MAP kinase pathway, having as main substrates MEK1 and 2 [66]. V600E mutation (BRAFV600E), characterized by the exchange of a valine for a glutamate, is the gene mutation most commonly found in this tumor and its oncogenic action is mediated by the constitutive activation of the Ras-Raf-Mek-ERK pathway [68].

*RET* gene encodes a membrane tyrosine kinase protein that is not expressed in thyroid cells physiologically [69]. However, the activation of RET in thyroid cells can occur due to a chromosomal rearrangement formed by the fusion of the intracellular tyrosine kinase domain, in the 3′ portion of the *RET* gene, with the 5′ termination of a heterologous gene, leading to the constitutive activation of a truncated form of RET, known as RET/PTC [70,71]. RET/PTC rearrangement leads to oncogenic actions mainly through the constitutive activation of the MAPK (RET/PTC-Ras-Raf-Mek-ERK) pathway [61,65].

Both BRAF and RET/PTC genetic alterations result in MAPK activation, even though they are associated to different phenotypes. Recently, a multiplatform analysis showed that BRAFV600E-positive tumors are correlated to lower differentiation scores when compared to PTCs harboring Ras mutations or RET/PTC translocations [72]. Moreover, a lower differentiation score, characterized by downregulation of genes involved in iodide metabolism such as NIS, Tg, TPO and DUOX, is consistent with the fact that BRAFV600E mutation is found at high frequency in metastatic iodine-refractory PTC [73].

Some studies suggest that the constitutive activation of the MAPK pathway plays an important role in the impairment of iodide-metabolizing gene expression during thyroid carcinogenesis [74,75,76,77].

Even though differentiated thyroid carcinoma is often a painless and curable disease, the cellular dedifferentiation can occur in about 5% of cases [78]. The cellular dedifferentiation can be a consequence of epithelial-mesenchymal transition (EMT), characterized by multiple biochemical and morphological changes enabling polarized epithelial cells to assume a mesenchymal phenotype, increasing proliferation, migration and invasiveness abilities, besides resistance to apoptosis [79,80]. The molecular basis of EMT is not completely elucidated.

Besides EMT, decrease or loss of expression of some thyrocyte differentiation markers, such as sodium-iodide symporter (NIS) and thyroglobulin (Tg), has been demonstrated in thyroid cancer [81]. Clinically, the dedifferentiation is characterized by the loss of the ability to uptake radioiodide, increasing aggressiveness of the tumor and reducing the efficacy of the radioiodine therapy [82]. Therefore, compounds able to retard or interrupt dedifferentiation and cancer progression could be very useful tools in thyroid carcinoma therapy. Flavonoids have been shown to possess anti-carcinogenic activity and thus are good candidates [1,83].

## 4. Flavonoids

**Figure 2:**
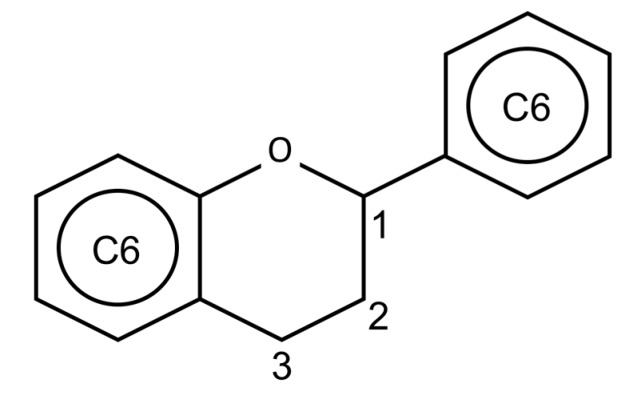
Basic chemical structure of the flavonoids.

Flavonoids are polyphenolic compounds widely spread in the vegetable kingdom. The basic chemical structure is C6-C3-C6 (Figure 2), composed of two aromatic rings linked by a three-carbon chain [84]. Most flavonoids are conjugated to carbohydrates, but they can also be found in a free form, known as aglycones [85]. Many biological actions are attributed to flavonoids in vegetables, among them we can cite: protection against ultraviolet and visible rays; protection against insects, fungi, viruses and bacteria invasion; attraction of insects to pollination; antioxidant effect and hormonal modulation [85].

The in vivo effect of a dietetic compound depends on its bioavailability. A study regarding the bioactivity and bioavailability of isoflavones and flavonoids revealed that they are influenced by the intestinal microflora [86]. Despite the appreciable degradation of these compounds in the intestines, 10% to 60% of the total reaches the plasma, and depending on the type and quantity of flavonoids in the diet, significant concentrations can be achieved. The amount of flavonoid available for absorption is also influenced by the processing and preparation of the food. For example, the isoflavone content in soybeans can be halved by cooking [86]. Thus, the application of in vitro data for in vivo situation should be considered, but with caution. Although some flavonoid metabolism may occur in peripheral tissues, most reactions seem to proceed in the digestive tube and in the liver, thus the levels in the target tissue are usually analogous to those in plasma. Some evidence indicates that the products of intestinal and hepatic metabolism of flavonoids and phytoestrogens can be biologically active too and mediate estrogen signaling, for example [87,88]. In conclusion, the great disparities in the effects of such compounds in humans can be due to differences in individual microflora, intestinal transit, and hepatic metabolism, among other factors, thus contributing to the inconsistency of the effects of flavonoids and phytoestrogens on humans [89,90].

## 5. Effect of Flavonoids on the Synthesis and Metabolism of Thyroid Hormones

In the beginning of the 1950s, Moudgal et al. demonstrated that rats fed a diet containing 20 mg of the glycosidic form of arachinoside and anacardioside, flavonoids isolated from nut pigments, developed goiter. These authors have shown that, these glycosides were able to inhibit thyroid iodide uptake, thus affecting thyroid hormone synthesis and leading to goitrogenesis [7]. In 1989, Gaitan et al. evaluated the effects of the consumption of Pearl Millet (*Pennisetum millet*), an important food consumed in poor regions of Africa and Asia, and observed that the consumption of this millet could have a goitrogenic effect in vivo and inhibit iodine organification in vitro [9]. Later, it was shown that two flavonoids (apingenin and luteolin) found in Fonio Millet (*Digitaria exilis*) have potent anti-thyroid effects, including the reduction of iodine organification and thyroid hormone secretion [10].

**Figure 3:**
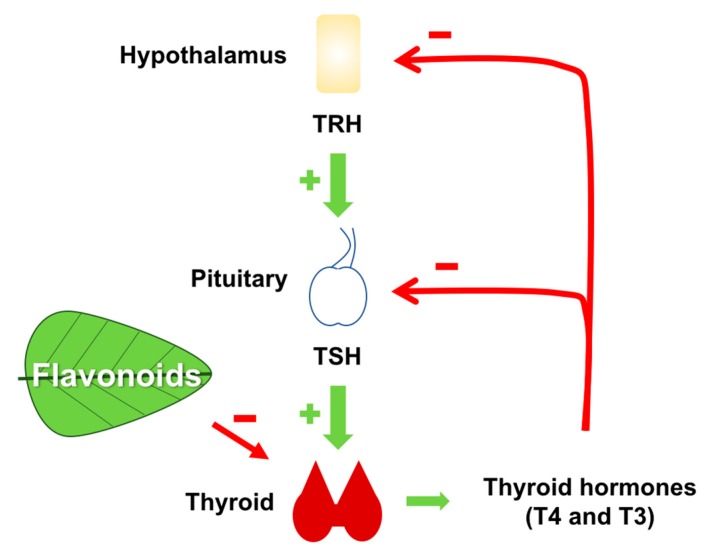
Some flavonoids can affect thyroid hormone synthesis. The inhibition of thyroperoxidase (TPO), the enzyme that catalyzes the synthesis of thyroid hormones, by some flavonoids can lead to reduction of thyroid hormone concentration in the serum, thus activating hypothalamus-pituitary-thyroid axis. Increased TSH then stimulates thyroid growth and can lead to goiter.

Notwithstanding, the mechanisms by which flavonoids could block thyroid hormones synthesis were not clear. However, another group of researchers performed two studies in which some flavonoids from the isoflavones group, found in large quantities in soybeans, were able to inhibit the synthesis of thyroid hormones by acting as alternative substrates for thyroperoxidase (TPO) [11,12]. TPO is the key enzyme in the synthesis of thyroid hormones [26,28]. Thus, the consumption of these flavonoids could lead to the reduction of thyroid hormone levels in serum, and consequently to an increase in TSH levels due to negative feedback mechanism (Figure 3). Increased TSH in turn would have a stimulatory effect on the thyroid, leading to hyperplastic and hypertrophic effects in the gland, leading to goiter. In fact, it has been shown that the consumption of soybeans could lead to a transient increase in TSH levels in humans, with a positive correlation between serum levels of daidzein (the main flavonoid found of soybean) and thyrotropin levels [91]. In a study performed by our group, the extract of *Kalanchoe brasiliensis*, a plant widely used in popular medicine in Brazil, known to be rich in flavonoids, was shown to inhibit TPO iodide oxidation activity by competitive mechanism, besides trapping H_2_O_2_ in vitro [13]. Since H_2_O_2_ is an essential cofactor for TPO activity, the effect of H_2_O_2_ trapping by *K. brasiliensis* extract could be responsible, at least in part, for the inhibition of TPO iodide oxidation activity [13]. Subsequently, we have shown that *Myrcia uniflora* (a plant popularly used for the treatment of diabetes mellitus) extract was also able to inhibit TPO activity in vitro. Two majority flavonoids were isolated and characterized in the extract of this plant: mearnsitrine and myricitrin, which were shown to be potent inhibitors of TPO [14].

**Figure 4:**
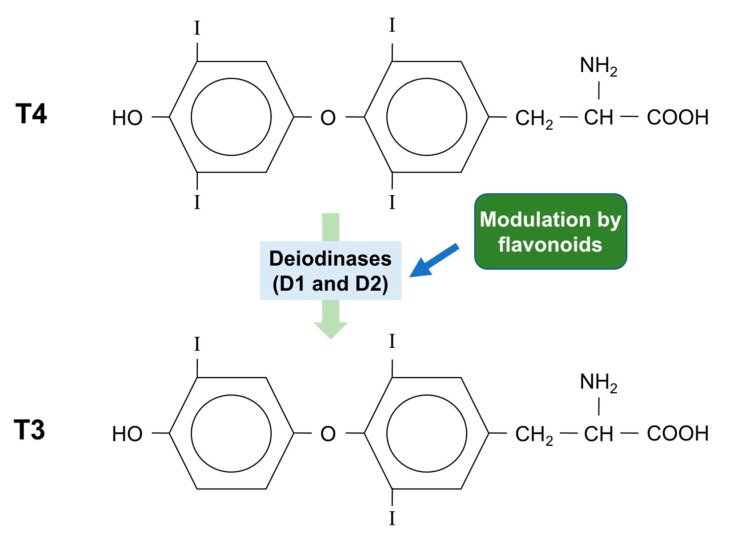
Some flavonoids can affect thyroid hormone metabolism. Type 1 (D1) and type 2 (D2) deiodinases catalyze the conversion of the pro-hormone T4 to the biologically active hormone T3. Some flavonoids affect this reaction by inactivating or activating deiodinases.

Even though the thyroid gland produces greater amounts of thyroxine (T4) than triiodothyronine (T3), the affinity of the thyroid receptor is 100 times higher for T3 than T4. T4 can be converted to T3 both in the thyroid and in peripheral tissue, by selecysteine-containing enzymes, known as iodothyronine deiodinases (Figure 4). There are three isoforms of deiodinase: type 1, type 2 and type 3 (D1, D2 and D3), and both D1 and D2 can convert T4 to T3 [92].

Our group has evaluated the in vitro effect of some flavonoids on thyroid type 1 deiodinase activity. All tested flavonoids (baicalein, quercetin, catechin, morin, rutin, fisetin, kaempferol and biochanin A) were able to inhibit, at least partially, deiodinase activity, however with differences in the inhibitory potency and in the mechanism of inhibition [17].

Synthetic flavonoids have also been shown to inhibit deiodinase activity. Spanka and coworkers, in 1990, have shown that the synthetic flavonoid EMD 21388 is a potent inhibitor of hepatic type 1 deiodinase activity in vitro [16].

Later, Da-Silva et al. have studied the effect of some flavonoids on type 2 iodothyronine deiodinase, using cell lines. The authors have shown that some flavonoids from the group of the flavonols increased type 2 deiodinase activity, with kaempferol having the greatest stimulatory effect, increasing ten times the enzymatic activity [18].

The effect of in vivo treatment with flavonoids on deiodinase activity has also been evaluated by our group and by others, with a predominantly inhibitory effect on D1 [15,19,20] and stimulatory effect on D2 [15].

## 6. Flavonoids and Cancer

Since cancer is a quite complex disease, with pathophysiology that varies greatly according to the cell type, the treatment has several difficulties and limitations. Depending on the kind of cancer, chemotherapy, surgery, hormone therapy and radiation are used in isolation or combination, aiming at the best results [93]. However, these treatments can lead to important side effects [94]. Therefore, the search for compounds able to prevent or treat cancer, leading to fewer side effects, has increased. Flavonoid administration has long been suggested to improve the tolerance to cancer treatment in an experimental model of cancer [95]. In addition, more recent studies suggest that flavonoids could be useful in the treatment of several types of cancer, such as skin, stomach, liver, lung and also thyroid cancer [93,96].

Epidemiological and pathological data suggest that thyroid cancer risk may be modified by flavonoids and phytoestrogen intake. Some phytoestrogens, such as those found in cruciferous vegetables, seem to be associated with a low risk of thyroid cancer [97]. On the other hand, soybeans have been associated with the development of goiter, which could be a risk factor for thyroid cancer [8,98]. In 2014, Xiao and collaborators published an epidemiological study, utilizing data from the NIH-AARP Diet and Health Study from 1995 to 1996. The authors observed that thyroid cancer risk was inversely associated with dietary flavan-3-ols, but positively associated with flavanones [99]. These epidemiological data suggest the important impact of dietary flavonoids on thyroid cancer and reinforce the potential of nutritional factors to the treatment of this disease. However, given the few studies in this field, additional research is still needed.

## 7. Effect of Flavonoids on Iodide Uptake and Thyroid Cancer

Although excessive intake of some flavonoids may be related to goiter, several studies have shown beneficial effects of these compounds for the thyroid cancer. Flavonoids were shown to have antiproliferative and cell re-differentiation effects, inducing the re-expression of NIS mRNA in an anaplastic thyroid carcinoma cell line (FRO), suggesting that these compounds could be important as therapeutic agents in the treatment of thyroid cancer [100,101,102]. In 2004, Schröder-van der Elst and colleagues evaluated the effects of some flavonoids on cell proliferation and NIS function and expression, using a follicular thyroid carcinoma cell line FTC-133, which does not express NIS and TPO, after transfection of these cells with human sodium iodide symporter cDNA (hNIS). Most of the tested flavonoids inhibited the growth of this tumor cell line, but also decreased radioiodide uptake. However, the flavonoid myricetin was able to significantly increase iodide content in the FTC-133 cells, possibly due to both increased iodide influx and decreased iodide efflux, besides reducing cell proliferation; thus suggesting that this flavonoid could be a promising therapeutic agent in the treatment of thyroid cancer [24]. In Table 1, we summarize some studies that evaluated the effect of flavonoids on different thyroid carcinoma cell lines.

Giuliani et al. studied the effects of the flavonoid quercetin on a rat thyroid cell line, FRTL-5. They have shown that quercetin treatment inhibited PI3K/Akt pathway, thus decreasing cell proliferation, but was also able to reduce mRNA levels of NIS gene, thus decreasing iodide uptake. It was suggested that the inhibition of phospholipase A2/lipoxygenase pathways could be the mechanism by which quercetin decreased NIS expression and iodide uptake [111]. Later, the same research group demonstrated that in vitro treatment with quercetin for 48 h was able to decrease the expression of proteins that are essential for thyroid hormone synthesis: thyroglobulin, thyroperoxidase, thyrotropin receptor and sodium-iodide symporter, in addition to their respective mRNAs. Additionally, the authors demonstrated that the administration of this flavonoid for 14 days (50 mg/kg BW, daily) to adult rats significantly reduced radioiodide uptake, although thyroid function was not affected, since serum levels of T3, T4 and TSH were not altered [112].

Our group evaluated the in vivo effect of the flavonoids biochanin-A, catequin, fisetin, morin, naringenin, naringin, quercetin and rutin on thyroid iodide uptake in rats. We have observed that, among the flavonoids tested, only the treatment with the flavonoid rutin was able to increase in vivo thyroid iodide uptake. Rutin administration for 5 days (20 mg/kg body weight, subcutaneously) was able to increase not only thyroid radioiodide uptake, but also NIS protein and mRNA levels in the thyroid of the animals. Even though TSH concentration remained unchanged in the serum of the rats treated with rutin, there was a significant increase in both protein and mRNA levels of thyrotropin receptor in thyroid, thus suggesting that the flavonoid could increase NIS directly or due to an increased responsiveness of the gland to TSH [15].

In 2014, a study suggesting a promising effect of the treatment with the flavonoid apingenin in combination with an inhibitor of Akt pathway was published, using the rat thyroid cell line PCCL3 as a model. In this study, the authors demonstrated that the previous inhibition of the Akt pathway followed by treatment with apingenin (20 μM) for 24 h was able to increase radioiodide uptake and that the p38MAPK pathway must be activated for this effect to occur [25]. Complementarily, an increase in the radioiodide uptake was observed in PCCL3 cell lines mutated to express BRAFV600E or RET/PTC oncogenes [113] and also in a primary thyroid tumor culture from TRβPV/PV genetically modified mice [25].

Celano et al. [114] evaluated the effect of flavonoids obtained from *Citrus reticulata* (mandarin) juice on anaplastic thyroid carcinoma cell lines. They have found that the cell number was reduced in the three cell lines tested, due to the arrest of cell cycle in G2/M. Additionally, the authors observed a reduction of cell migration, which seems to be related to a reduction in the activity of a metalloproteinase.

An interesting study evaluated the effect of quercetin, delivered from a hydrogel of hyaluronic acid, in association with an inhibitor of Aurora Kinase, on medullary and papillary thyroid cancer cell lines. The authors have found that quercetin had an anti-inflammatory action and also that the compounds had a synergic cytotoxic effect, thus suggesting the anti-tumor activity of the substances [115].

Therefore, data from literature reinforce the idea that many flavonoids, and maybe the association among them, could be useful in the therapy of thyroid cancer, both increasing radioiodide uptake and reducing tumor growth.

## 8. Conclusions

In summary, there are literature data showing that flavonoids can affect many parameters in thyroid cancer, including cell proliferation, invasiveness and differentiation. Moreover, some flavonoids were shown to stimulate iodide uptake, a fundamental step for thyroid cancer therapy. Therefore, these phytochemicals have a great potential as therapeutic agents both as adjuvant in radioiodine therapy and to limit tumor growth and invasiveness. Indeed, additional studies are needed to better clarify the mechanisms underlying the effect of the flavonoids on thyroid cancer and the possible side effects associated with the pharmacological use of these compounds, to enable the development of drugs that could be useful in the treatment of thyroid cancer.

## Figures and Tables

**Table 1:** Effect of some flavonoids on thyroid cancer.

Reference	Experimental Design	Summary of Results
Schröder-van der Elst et al., 2004 [24].	Follicular thyroid cancer cell line (FTC-133) was treated with different flavonoids and ^125^I uptake, ^125^I efflux and DNA content of the cells were measured.	Most flavonoids inhibited cell growth. Myricetin was the only flavonoid studied that increased the influx and decreased the efflux of ^125^I.
Liu et al., 2004 [103].	Human medullary carcinoma cell line (TT) was treated with a Src-specific tyrosine kinase inhibitor, PP2, or genistein and cell proliferation was examined.	Compared to control, genistein caused a modest decline in cell count and DNA synthesis, with minimal changes in apoptosis.
Phan et al., 2011 [102].	HTH7 and KAT18 cells, derived from patients with anaplastic thyroid cancer (ATC), were treated with chrysin for up to 6 days.	Chrysin reduced ATC cell numbers by increasing apoptosis in vitro.
Kang et al., 2011 [101].	Authors evaluated the effect of polyphenols (resveratrol, genistein, quercetin, kaempferol and resorcinol) on cell growth and NIS expression in thyroid cancer cell lines (TPC-1—papillary thyroid cancer; FTC-133—follicular thyroid cancer; NPA—poorly differentiated papillary thyroid cancer; FRO—undifferentiated/anaplastic thyroid cancer and ARO—undifferentiated/anaplastic thyroid cancer).	Growth of thyroid cancer cell lines was inhibited in response to genistein, resveratrol and quercetin. NIS mRNA increased in FTC-133 cells in response to genistein and resveratrol but there was no change in NPA, FRO and ARO cells. Quercetin induced NIS in FTC-133, NPA and FRO cells.
Lim and Cha, 2011 [104].	Human ATC cell line, ARO, was treated with epigallocatechin-3-gallate (EGCG).	EGCG inhibited cell proliferation and induced apoptosis via suppression of the EGFR/ERK pathway and cyclin B1/CDK1 complex in ATC cells.
Ahn et al., 2012 [105].	The effect of photodynamic therapy (PDT) and genistein was studied in a human anaplastic thyroid cancer cell line (SNU 80).	The individual treatment with PDT induced apoptosis in SNU 80 cells; however, the efficacy was greatly increased by association with genistein.
Mazumdar et al., 2013 [106].	A human MTC cell line, TT, was incubated with theaflavins, the bioactive components of black tea.	Theaflavins induced apoptosis in human MTC cell line by downregulating both PI3K/Akt/Bad and Ras/Raf/ERK pathways.
Yu et al., 2013 [107].	The effect of chrysin on tumor growth was evaluated using both in vitro model (ATC cell lines: HTh7 and KAT18) and in vivo using subcutaneous xenograft tumor model.	Chrysin inhibited tumor growth in ATC both in vitro and in vivo, which seems to be due to Notch1 signaling activation, leading to cancer cell apoptosis.
De Amicis et al., 2013 [108].	Authors investigated the effect of EGCG, a major catechin in green tea, on the proliferation and motility of human thyroid papillary (FB-2) and follicular (WRO) carcinoma cell lines.	Epigallocatechin-3-gallate reduced proliferation of both thyroid cancer cell lines, besides decreasing cell motility and migration. Those effects seem to be mediated by loss of epithelial-to-mesenchymal transition markers.
Kim et al., 2013 [109].	Authors studied the effect of apigenin on anaplastic thyroid carcinoma cell line (FRO) survival and c-Myc expression.	Apigenin induced apoptosis via c-Myc increment, along with increased phosphorylation of p53 and p38 in FRO cells.
Patel et al., 2014 [110].	The effect of hesperetin on an ATC cell line (HTh7) proliferation and differentiation was evaluated.	Hesperetin reduced ATC cell proliferation and induced the expression of thyroid markers, including sodium-iodide symporter.
Zhang et al., 2015 [23].	A papillary thyroid cancer cell line (BCPAP) was treated with apigenin and the effect on cell viability and the underlying mechanisms were studied.	Apigenin treatment reduced cell viability by inducing ROS generation, leading to DNA damage and a subsequent cell cycle arrest in G2/M phase. Autophagy was induced and eventually triggered human papillary thyroid cancer cell death.
Mutlu Altundag et al., 2016 [22].	Human papillary thyroid cancer cells (BCPAP) were treated with quercetin and cell viability and apoptosis were evaluated.	Quercetin induced reduction of BCPAP cell viability by enhancing apoptosis. This effect seems to involve downregulation of HSP90, a heat shock protein important for stress response in cancer cells.

FTC-133—Follicular thyroid cancer cell line; TT—Human medullary carcinoma cell line; PP2—Src-specific tyrosine kinase inhibitor; ATC—anaplastic thyroid cancer; HTH7 and KAT18—anaplastic thyroid cancer cells derived from patients with ATC; NIS—sodium-iodide symporter; TPC-1—papillary thyroid cancer; NPA—poorly differentiated papillary thyroid cancer; FRO—undifferentiated/anaplastic thyroid cancer and ARO—undifferentiated/anaplastic thyroid cancer; EGCG—epigallocatechin-3-gallate; PDT—photodynamic therapy; SNU 80—human anaplastic thyroid cancer cell line; FB-2—human thyroid papillary carcinoma cell line; WRO—human thyroid follicular carcinoma cell line; BCPAP—papillary thyroid cancer cell line.
